# Affinity-selected heparan sulfate collagen device promotes periodontal regeneration in an intrabony defect model in Macaca fascicularis

**DOI:** 10.1038/s41598-023-38818-y

**Published:** 2023-07-21

**Authors:** Xiaoman Luo, Chau Sang Lau, Bach Quang Le, Tuan Chun Tan, Jian Hui Too, Raymond Alexander Alfred Smith, Na Yu, Simon M. Cool

**Affiliations:** 1 Institute of Molecular and Cell Biology, Agency for Science, Technology and Research (A*STAR), 61 Biopolis Dr, Proteos, Singapore, 138673 Singapore; 2 National Dental Research Institute Singapore, National Dental Centre Singapore, 5 Second Hospital Ave, Singapore, 168938 Singapore; 3 Duke-NUS Medical School, National University of Singapore, Singapore, 169857 Singapore; 4 Bioprocessing Technology Institute, Agency for Science Technology and Research (A*STAR), 20 Biopolis Way, Singapore, 138668 Singapore; 5 School of Chemical Engineering, The University of Queensland, 46 Staff House Rd, St Lucia, QLD 4072 Australia

**Keywords:** Dental diseases, Biomaterials, Biomimetics

## Abstract

It is challenging to regenerate periodontal tissues fully. We have previously reported a heparan sulfate variant with enhanced affinity for bone morphogenetic protein-2, termed HS3, that enhanced periodontal tissue regeneration in a rodent model. Here we seek to transition this work closer to the clinic and investigate the efficacy of the combination HS3 collagen device in a non-human primate (NHP) periodontitis model. Wire-induced periodontitis was generated in ten Macaca fascicularis, and defects were treated with Emdogain or collagen (CollaPlug) loaded with (1) distilled water, (2) HS low (36 µg of HS3), or (3) HS high (180 µg of HS3) for 3 months. At the endpoint, microscopic assessment showed significantly less epithelial down-growth, greater alveolar bone filling, and enhanced cementum and periodontal ligament regeneration following treatment with the HS-collagen combination devices. When evaluated using a periodontal regeneration assessment score (PRAS) on a scale of 0–16, collagen scored 6.78 (± 2.64), Emdogain scored 10.50 (± 1.73) and HS low scored 10.40 (± 1.82). Notably, treatment with HS high scored 12.27 (± 2.20), while healthy control scored 14.80 (± 1.15). This study highlights the efficacy of an HS-collagen device for periodontal regeneration in a clinically relevant NHP periodontitis model and warrants its application in clinical trials.

Periodontitis is one of the most common chronic inflammatory diseases that affects the periodontium, involving multiple tooth-supporting tissues, including cementum, periodontal ligament (PDL) and alveolar bone^1^. The inflammation of the periodontium, mainly caused by the inflammatory response to bacterial accumulation on the teeth, can contribute to the progressive destruction of collagenous and bony tissues around the teeth, eventually causing the loosening and premature loss of teeth^2^. The consequences of periodontitis are severe, with the loss of periodontal tissues and teeth impacting oral function, nutritional intake, and general well-being. Periodontitis is the primary cause of tooth loss in adults and has a high prevalence worldwide, with severe periodontitis affecting 10.8% or 743 million people aged 15–99 years globally^3^. In Southern Latin America, the prevalence of severe periodontitis is as high as 20.4%^4^. The inflammatory responses triggered by periodontitis can lead to further health problems, such as the exacerbation of diabetes and increased risk of cardiovascular disease and premature low birth weight^5,6^.

The treatment of periodontitis currently focuses on removing bacterial accumulation and inhibiting further inflammation and tissue loss via scaling, root planning, open-flap debridement, and establishing positive oral hygiene^7^. However, such treatments do not restore the damaged periodontal tissues. The limited regenerative potential of the periodontium, especially cementum and PDL, poses significant challenges in periodontal regeneration^8^. In periodontal defects where the tooth root is exposed to the oral cavity, rapidly proliferating fibrous and epithelial cells could infiltrate the defect site and disrupt the sequential reconstruction of the cementum, PDL and alveolar bone, leading to residual periodontal defects that are prone to future tissue breakdown and reoccurrence of periodontitis^9^.

Current approaches to restoring the periodontium include guided tissue regeneration (GTR), bone grafting, application of growth factors, and using an enamel matrix derivative (EMD)^10^. The principle of GTR is the use of a physical barrier to exclude epithelial and gingival connective tissue infiltration into the periodontal defect and creating a preferential space to cells that regenerate the cementum, PDL and alveolar bone^11^. GTR shows highly variable results in lateral and vertical periodontal tissues, is technically challenging to perform and carries a risk of barrier breaching and subsequent bacterial infections^12,13^. Bone grafting, the implantation of bone harvested from the host or another human/animal, is effective in regenerating alveolar bone. However, the harvest of bone from the host leads to additional surgical procedures and morbidities, while bone grafts derived from another human/animal may pose risks of disease transmission and immune rejection^14,15^.

Applications of growth factors provide a therapeutic option for dental regeneration to improve clinical outcomes^16^. Bone morphogenetic protein-2 (BMP2), fibroblast growth factor-2 (FGF2) and platelet-derived growth factor (PDGF) have been combined with synthetic bone grafts to enhance regeneration of the damaged tissues^17–19^ Our group has previously demonstrated the potential of BMP2 and FGF2- containing devices in the regeneration of periodontal tissues in both rat and NHPs models and have observed significant improvement in epithelial down growth, cementum, and ligament regeneration^20^. A recent randomized pilot trial also demonstrated the efficacy of BMP2 with PLA/PGA membrane on periodontal regeneration in Class II mandibular furcation defects in 24 patients^21^. However, growth factor treatment is expensive and plagued by safety concerns, as supraphysiological doses are required that may be associated with medical complications such as ectopic bone formation and radiculopathy^22^ that can result in life-threatening adverse events^23,24^. Moreover, their short half-life and sensitivity to storage hinder their ability to deliver reliable and cost-effective treatment efficacy for regenerative procedures.

The use of enamel matrix derivative (EMD), an extracellular matrix derivative extracted from porcine tooth buds, is gaining popularity in periodontal treatments due to its efficacy in periodontal regeneration^25^. The most commonly applied EMD product is Emdogain (Straumann, Basel, Switzerland), which is commercially available to treat intrabony periodontal defects^26–28^. Despite its commercial success, EMD delivers variable clinical outcomes, and the mechanism of action is poorly understood due to its complex mixture of proteins^29^. The purification of EMD from developing porcine enamel matrix makes EMD susceptible to batch-to-batch variations like most naturally derived materials^30^. Moreover, the protein-based composition of EMD can lead to disadvantages associated with growth factors, i.e. high costs and growth factor-related safety concerns.

Due to the limitations of current therapies, there is interest in developing innovative solutions that can effectively, economically, reliably, and safely achieve periodontal regeneration. Here, we present a glycosaminoglycan-based strategy to enhance the regenerative activities of endogenous growth factors that avoids the need for supraphysiological dosing of exogenous growth factors. Glycosaminoglycans, like heparan sulfate (HS), are among the most critical components in the extracellular matrix and are known for their ability to bind and modulate growth factors^31,32^. They can be used as protein cargos or engineered as functionalized scaffold materials that can bind endogenous growth factors, protect them from enzymatic degradation and promote their activities. Among the many variants of HS, our group has developed a BMP2-binding HS variant (termed HS3) with enhanced osteostimulatory activity in vitro and in vivo^33–38^. Briefly, HS3 can bind to BMP-2, and increase its bioavailability, bioactivity and half-life^33^. Notably, HS3 increased bone formation and scaffold integration in a rat calvarial defect model without exogenous BMP2^37^.

We have previously investigated the efficacy of HS3-collagen devices on the regeneration of periodontal tissues in surgically created periodontal defects in rodents^39^. We found that HS3 promoted bone regeneration and functional ligament restoration. Moreover, HS3/BMP2 combinations improved the regeneration of periodontal tissues and reduced epithelial down-growth. However, surgically created defects in healthy alveolar ridges in rodents do not recapitulate the bacterial-induced inflammatory pathogenesis of periodontitis. Also, there are known differences in healing rates, defect sizes and oral flora between rodents and humans^40^. In comparison, NHPs’ possess mandible size, dentition, and periodontal anatomy that align more closely with humans^41^. This not only enables the creation of clinically relevant mandibular defects that can be standardized and reproduced but also allows repetitive non-invasive clinical examinations to monitor healing and plaque, similar to real clinical settings^20^. Moreover, the similarity of oral microbial pathogens (*P. gingivalis*) between NHPs are similar to humans^42^, and the possibility to place ligatures in NHPs to promote plaque formation enable the creation of experimental microbiological and inflammatory conditions that closely approximate challenges in human periodontitis. With a level of clinical relevance that is unmatched by other animal models, NHP models have been used extensively to study periodontal diseases and periodontal regeneration^41,43^. We have also demonstrated the effectiveness of the NHP periodontitis model in our previous study^20^. Hence, we find the NHP model highly important for the evaluation of our HS3-collagen devices before we can move forward to clinical implementation.

The present study aims to assess the efficacy of HS3-collagen devices for periodontal regeneration in a wire-induced inflammatory periodontal defect model in NHPs. As this is the first efficacy study using HS3-collagen devices in NHPs and the effective dose in this model remains unclear, two types of HS3-collagen devices containing different amounts of HS3 (HS low = 36 µg; HS high = 180 µg) loaded onto Collaplug (Zimmer Biomed, Warsaw, IN, USA) cylinders were evaluated and compared with collagen alone (Collaplug cylinders without additives) and Emdogain (Straumann, Basel, Switzerland). Collaplug, a hemostatic collagen sponge commonly used in oral surgical procedures^44^, served as the negative control and a carrier for HS3. Emdogain, which is currently an established option for periodontal treatment^26,45,46^, served as a positive control. Since this study aims to use HS3 to enhance the activities of BMP2 produced by the host, and the effects of exogenous BMP-2 have been already well-reported, BMP2 treatment is not included in this study. We hypothesized that the HS groups would have similar or better efficacy in periodontal regeneration as the Emdogain group, and the null hypothesis would be the HS groups showing similar efficacy as the Collagen group.

## Results

### Animal usage and sample processing

**Figure 1:**
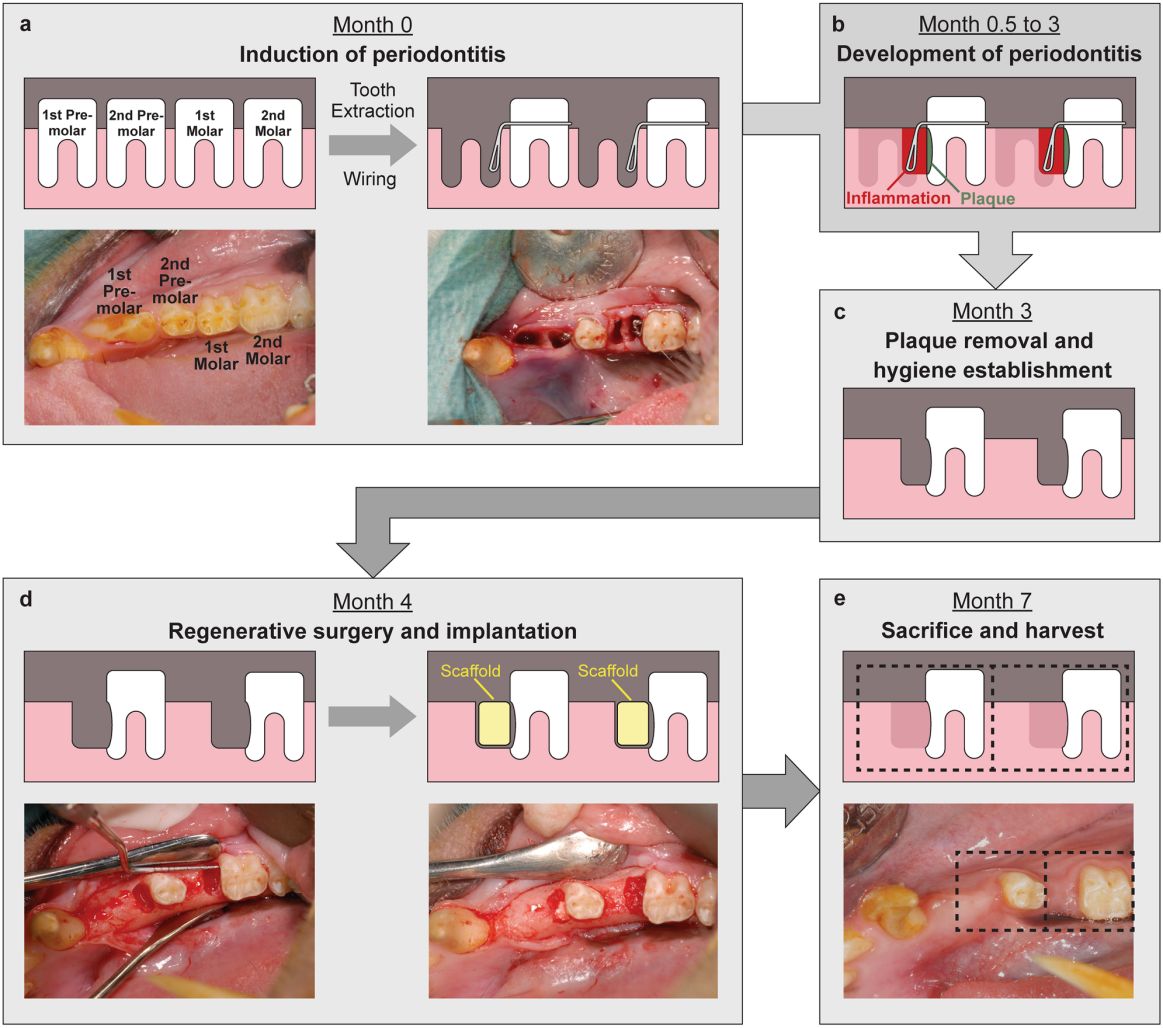
Schematic overview of the surgical model and experimental procedures (**a**): Induction of periodontitis at month 0; (**b**): Development of periodontitis till month 3; (**c**): Plaque was removed and oral hygiene established at month 3; (**d**): Regenerative surgery and implantation of the scaffolds at month 4; (**e**): animals were sacrificed at month 7, tissues framed by the dashed lines were harvested for analyses.

All animals tolerated the surgical procedures and remained healthy throughout the study. Of the 40 defects created in 10 animals, 32 were used in the current study and the rest in a parallel study according to animal reduction principles and ethics^47^. Of the 32 samples, one was damaged during histological sectioning (Col group, animal ID 7330, right molar), and no complete tissue samples were able to be retrieved. Another specimen was sectioned at the oblique plane towards the root axis, hence no valid histological measurement was obtained (HS high group, animal ID 8348, left premolar). After viewing the histological sections, one sample was diffused with lymphocytes associated with food trapping that traumatized the gingiva (HS low group, animal ID 816, left premolar). These three samples were, therefore, not included in further analyses. Therefore 29 samples were included in the histological analysis, of which 9 samples were from the Collagen group, 5 samples were from Col + HS Low group, 11 samples were from Col + HS High group, and 4 samples were from the Emdogain group. Detailed procedures and important events of the animal study are shown in Fig. 1. Detailed animal usage and sample processing are indicated in Supplementary Table 1.

### Clinical assessment of the NHP model

**Figure 2:**
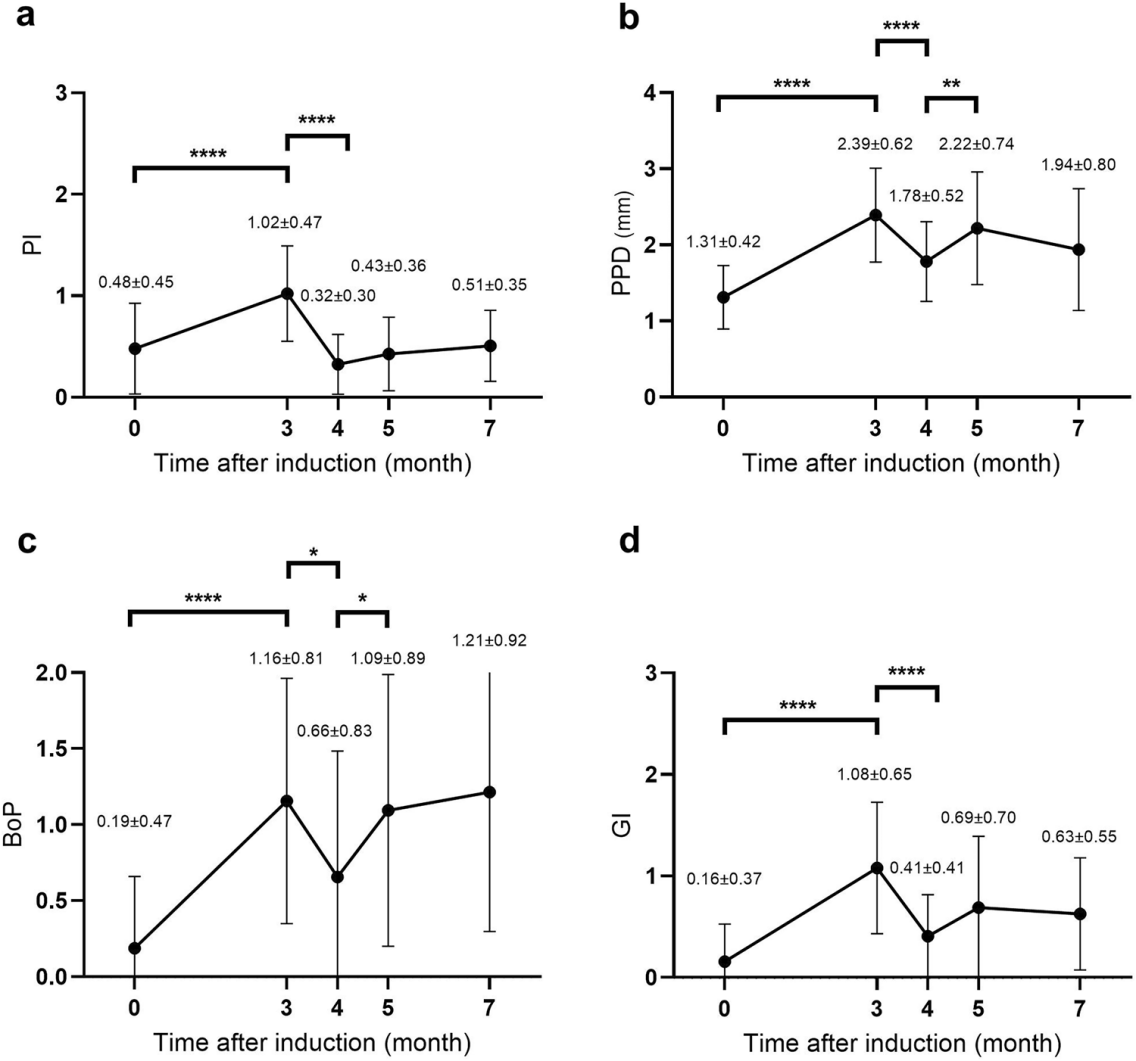
The four indexes used for clinical assessments during the experiment period. (**a**): Increased plaque index. (**b**): Periodontal probing depth. (**c**): Number of bleeding points on probing. (**d**): Gingival inflammation index. Important events at each time point: Month 0—prior to tooth extraction and wiring; Month 3—prior to wire removal and scaling; Month 4—prior to scaffold implantation; Month 5—1 month post plug implantation; Month 7—3 months post scaffold implantation.

After extraction of the first premolars and first molars and the introduction of wires at Month 0, symptoms of periodontitis were observed, as indicated by the increase of plaque index (PI), periodontal probing depth (PPD), number of bleeding points on probing (BoP) and gingival inflammation index (GI) observed at Month 3 (*p* < 0.0001, Fig. 2). After plaque control and the establishment of oral hygiene, the scale of all 4 parameters decreased at Month 4 (PI: *p* < 0.0001; PPD: *p* < 0.0001; BoP: *p* = 0.017; GI: *p* < 0.0001) and almost returned to the baseline level. The clinical parameter changes from Month 0 to Month 4 were similar to those observed in patients receiving initial periodontal treatments. At month 5 (1 month after the regenerative surgery), a mild inflammatory response was observed, as indicated by an increase in the 4 indices (PI: *p* = 0.214; PPD: *p* = 0.008; BoP: *p* = 0.046; GI: *p* = 0.056). The slight inflammatory responses (BoP and GI) at Month 5 subsided at Month 7, indicating soft tissue had mainly recovered without any chronic inflammation. Together, these observations suggested successful simulation of the conditions for periodontitis in this NHP model.

### Radiographic observation

**Figure 3:**
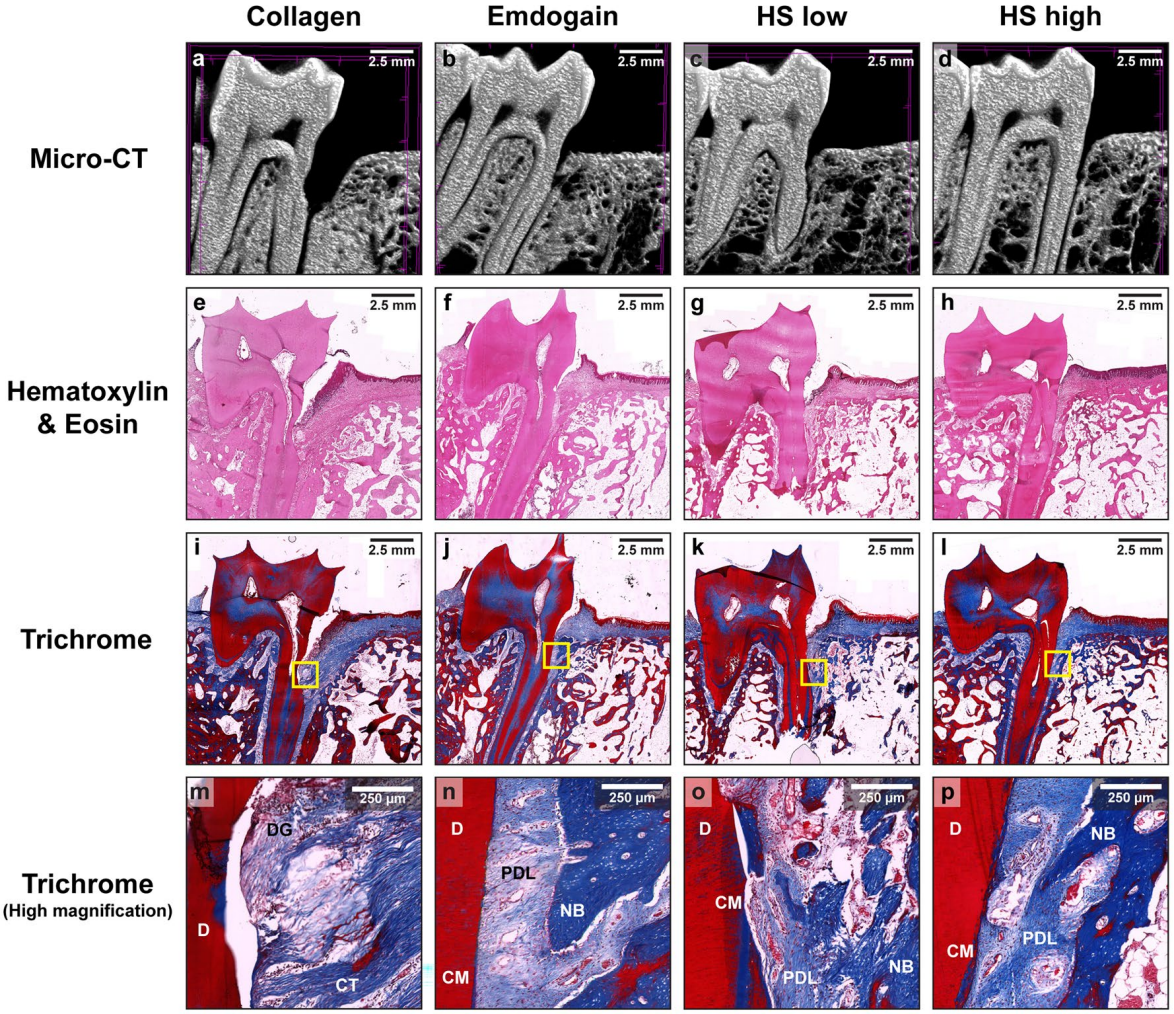
Representative images of micro-CT cross-sections, Hematoxylin & Eosin stained sections and Trichrome stained sections. The framed area indicated in the Trichrome stained sections are presented at a higher magnification below the Trichrome images. CM, cementum; CT, connective tissue; D, root dentin; NB, new bone; PDL, periodontal ligament.

Bone regeneration in the defect area was evident in micro-CT imaging, especially for Emdogain, HS low and HS high groups (Fig. 3). Notably, new bone formation had rebuilt most of the lost alveolar bone surrounding the tooth in these groups, but not for the Collagen group. For micro-CT data, we observed that the newly formed bone showed similar attenuation to the host bone, making it challenging to identify the exact boundary of the original defect site. Therefore, it is impractical and inaccurate to obtain quantitative measurements of the extent of new bone formation inside the defect area solely based on the micro-CT data.

### Histological observation

The histological sections stained with H&E and trichrome offer a more detailed illustration of the extent of bone regeneration and the tissue response to various treatment implants (Fig. 3). The implants were no longer visible in the defect area for all groups, suggesting complete degradation and resorption. New bone tissue was observed in the defect area of the Emdogain group (Fig. 3f,j), HS low group (Fig. 3g,k) and HS high group (Fig. 3h,l), while the defect area in the Collagen group was largely occupied with fibrous connective tissue and limited formation of new bone (Fig. 3e,i). The bone tissues in the Emdogain and HS high groups were dense and mature (Fig. 3j,l). Obliquely oriented periodontal ligament was observed in the Emdogain, HS low and HS high groups, leaving only a small gap between the alveolar bone and the tooth (Fig. 3n–p). A longer and more continuous deposited cementum layer was observed in the Emdogain and HS high groups (Fig. 3n,p). In contrast, a layer of regenerated cementum could only be observed on the root surface close to the apical extent in the HS low group (Fig. 3o). The invasion of epithelial downgrowth towards the apical extent along the root surface was observed in the Collagen group, with limited cementum formation (Fig. 3m). No signs of severe inflammation or granulation tissues were observed in all analyzed sections, indicating an absence of chronic inflammation in the analyzed specimens.

### Regeneration of alveolar bone

**Figure 4:**
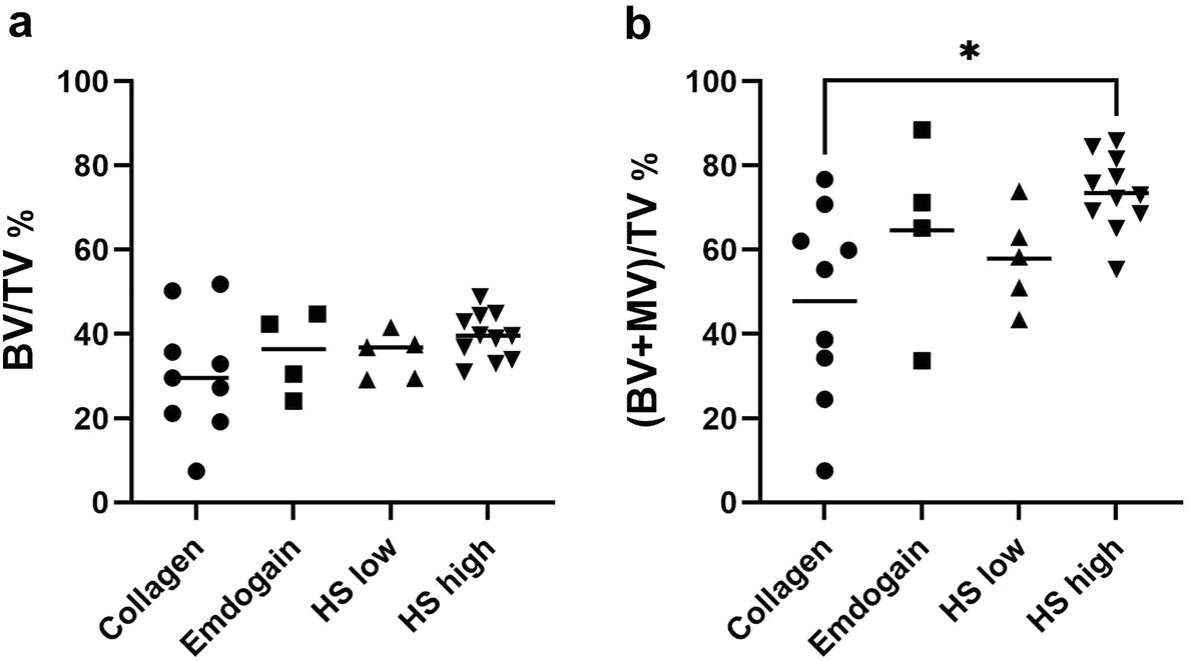
Bone formation in the ROI of the 4 treatment groups based on quantitative histomorphometry. (**a**): BV/TV%, the percentage of bone trabeculae formed in the ROI (as indicated in Fig. 7) 3 months after the implantation, excluding the bone marrow between bone trabeculae; (**b**): (BV + MV)/TV%, the percentage of bone tissue in the ROI including bone marrow between bone trabeculae 3 months after the implantation.

Compared to µCT imaging, histological sections showed more details in soft tissues that can be used as landmarks to define a standardized ROI for quantitatively measuring new bone formation in the defects. All defects were filled with either fibrous tissue or newly formed cancellous bone and bone marrow. Bone histomorphometry showed highly variable bone regeneration in the Collagen group (Fig. 4). The mean (± SD) volume of bone trabeculae (excluding the bone marrow) in the ROI was 30.6% (± 14.3%) for collagen alone, 35.4% (± 9.7%) for Emdogain, 34.9% (± 5.4%) for HS low, and 39.4% (± 5.5%) for HS high group (Fig. 4a). High variability in bone filling was observed in the Collagen group (co-efficient of variation (CV) = 46.7%) compared to Emdogain (CV = 27.5%), HS low (CV = 15.4%) and HS high group (CV = 13.9%). When the volume of bone marrow was included, the filling of cancellous bone in the ROI was 47.8% (± 22.9%) for collagen, 64.6% (± 22.9%) for Emdogain, 57.9% (± 11.6%) for HS low, and 73.5% (± 9.0%) for HS high. HS high group showed significantly higher filling than the Collagen group (*p* = 0.012, Fig. 4b).

### Histomorphometry of DG, CM, PDL and BA

**Figure 5:**
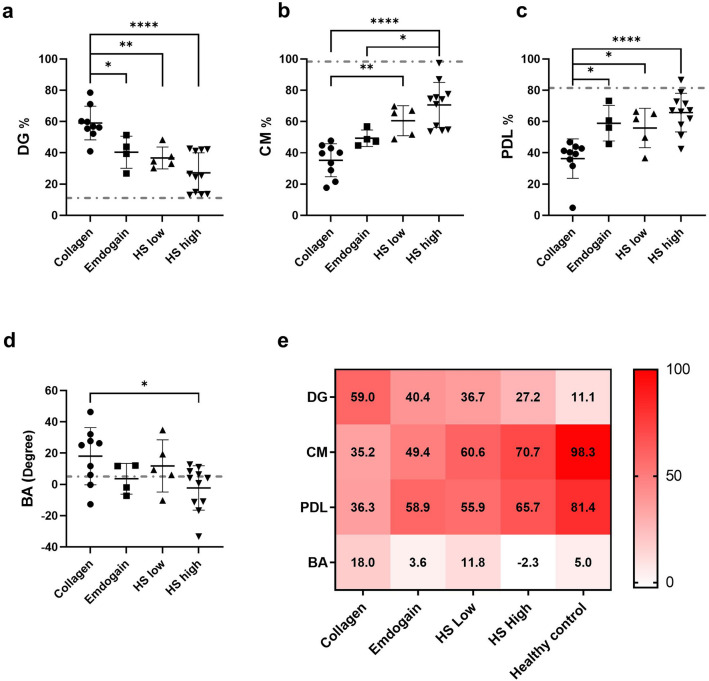
Microscopic measurements of epithelial downgrowth (DG), regenerated cementum (CM), regenerated periodontal ligament (PDL) and bone angle (BA) of the 4 treatment groups 3 months after the implantation. (**a**): Epithelial downgrowth; (**b**): Regenerated cementum; (**c**): Regenerated periodontal ligament; (**d**): Bone angle. Horizontal dashed lines in (**a**–**d**) represented the mean value of healthy control. (**e**): Result of the 4 measurements summarized in a heat map. The mean value of each measurement was given in the grids. Values obtained from healthy control also included for comparison.

Treatment with collagen alone resulted in a mean (± SD) epithelial downgrowth of 59.0% (± 10.8%) (Fig. 5a), while Emdogain or HS treatments reduced the mean epithelial downgrowth significantly to 40.4% (± 10.3%) (Emdogain, *p* = 0.044), 36.7% (± 7.0%) (HS low, *p* = 0.007) and 27.2% (± 12.7%) (HS high, *p* < 0.0001).

The mean (± SD) cementum regeneration (CM) was limited to 35.2% (± 10.5%) in the collagen group but was improved slightly to 49.4% (± 5.3%) with the treatment of Emdogain (*p* = 0.156) and improved significantly to 60.6% (± 9.6%) (*p* = 0.003) and 70.7% (± 14.4%) (*p* < 0.0001) in the presence of low and high doses of HS respectively (Fig. 5b). Notably, HS high also outperformed Emdogain significantly in CM (*p* = 0.018). Meanwhile, the collagen group’s mean (± SD) periodontal ligament regeneration (PDL) was limited to 36.3% (± 12.6%) but improved significantly with Emdogain or HS. The PDL regeneration increased to 58.9% (± 11.4%) for Emdogain (*p* = 0.026), 55.9% (± 12.6%) for HS low (*p* = 0.041) and 65.7% (± 12.3%) for HS high (*p* < 0.0001) (Fig. 5c).

Treatment with collagen alone led to a mean (± SD) BA of 18.0° (± 18.3°), while the addition of Emdogain and HS low reduced the BA to 3.6° (± 9.8°) and 11.8° (± 16.7°), respectively (Fig. 5d). These differences were however not statistically significant. The addition of HS high significantly reduced the BA to -2.3° (± 14.2°) (*p* = 0.036).

Compared to collagen control, Emdogain, HS low, and HS high resulted in DG, CM, PDL, and BA measurements closer to healthy control(Fig. 5e).Notably, none of the experimental groups’ mean value has achieved the level of the healthy control 3 months after regenerative surgery (Fig. 5a–e).

Periodontal regeneration assessment score (PRAS).

PRAS scores showed that on a scale of 0–4, healthy controls averaged 3.73 (± 0.46) for DG, 4.00 (± 0.00) for CM, 3.53 (± 0.52) for PDL and 3.53 (± 0.52) for BA (Supplementary Fig. 1a-d). The average total score of healthy controls was 14.80 (± 1.15), with the lowest score being 13 and the highest score being 16 (Supplementary Fig. 1e). According to the scoring rubric, all 15 healthy samples fall within the “normal” range (total score = 13–16). This validated the scoring system and the health status of the periodontal site, at least for the “normal range”.

**Figure 6:**
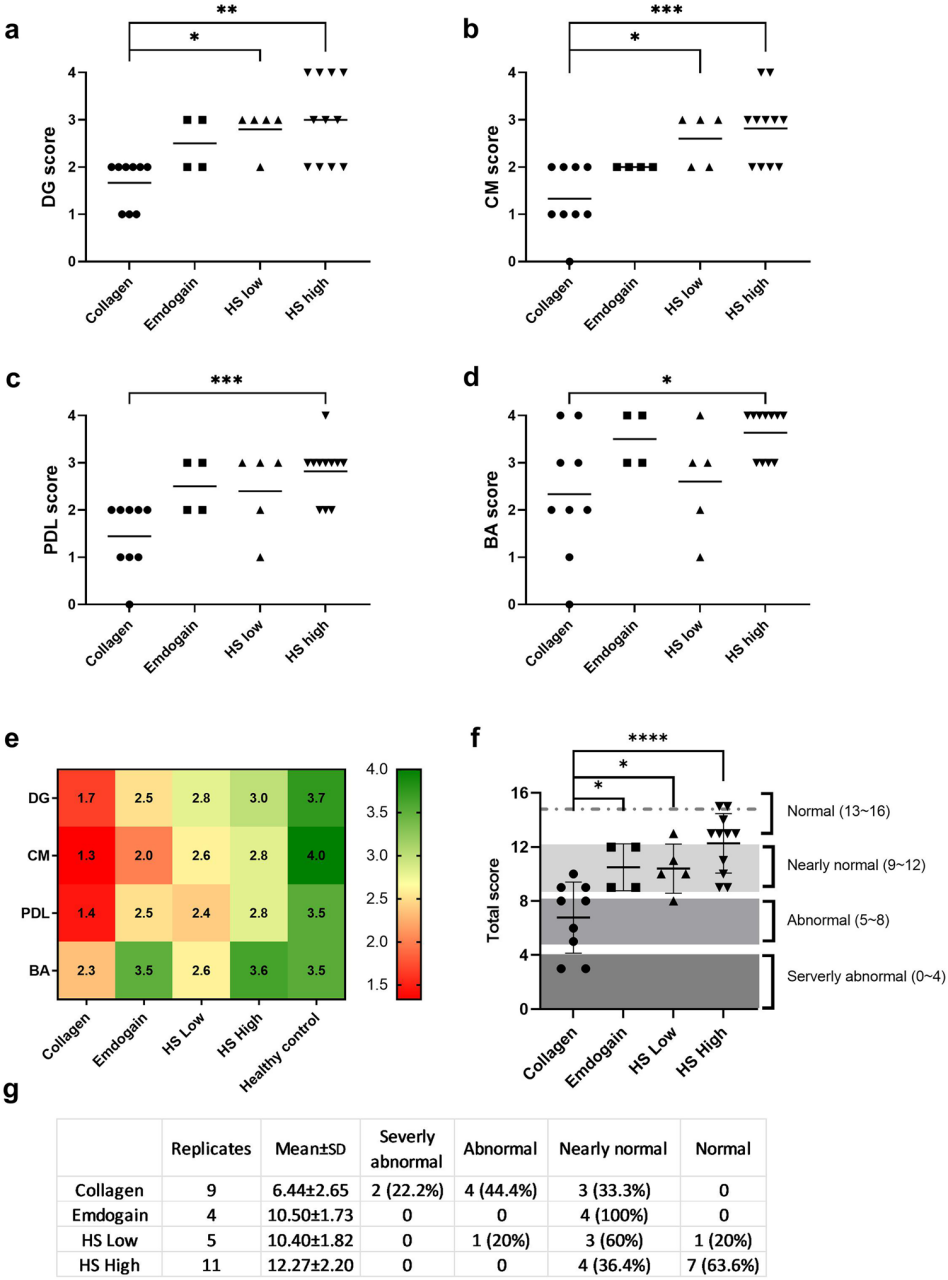
Periodontal regeneration assessment score (PRAS) of the treatment groups 12 weeks after the implantation. (**a**): DG score; (**b**): CM score; (**c**): PDL score; (**d**): BA score; (**e**): The 4 scores of the treatment groups presented in a heat map, and compared with healthy control. (**f**): The total PRAS scores; (**g**): A summary of the PRAS total scores and the distribution of grades.

We then applied the PRAS scoring system to the experimental groups. The individual scores showed similar trends to the microscopic measurements (Fig. 6a-d). When converted to a heatmap (Fig. 6e), the data shows improved periodontal regeneration with all three treatment groups (Emdogain, HS low, and HS high) as all four scores were elevated towards the healthy control level and higher than the Collagen group. The greatest improvement was shown in HS high, where all the BA scores were equivalent to healthy controls (3.6 (± 0.5) vs. 3.5 (± 0.5)), while DG, CM, and PDL scores were about 70%-80% of the level of healthy controls.

The mean (± SD) PRAS total score of the Collagen group is 6.78 (± 2.64). A significant improvement in the total score was seen when the defects were treated with Emdogain (10.50 (± 1.73), *p* = 0.049), HS low (10.40 (± 1.82), *p* = 0.037) or HS high (12.27 (± 2.20), *p* < 0.0001) (Fig. 6f–g). The mean values of the Emdogain and HS low groups were 1.6 times higher, and the mean value of the HS high group was 1.9 times higher than that of Collagen group. The total scores were not statistically significant amongst Emdogain, HS low and HS high groups though (HS low vs Emdogain *p* = 0.999, HS high vs Emdogain *p* = 0.539, HS low vs HS high *p* = 0.426). In Collagen group, two-thirds of the samples were rated “Abnormal” or “Severely abnormal”, one-third were rated as “Nearly normal”, and none was rated “Normal” (Fig. 6g). When treated with Emdogain, 100% of the samples achieved a “Nearly normal” rating. Notably, a “Normal” rating was only observed with HS groups, which is 1 out of 5 (20%) for the HS low and 7 out of 11 (63.6%) for the HS high group. HS high was the only treatment that repaired more than half of the defects to a “normal” rating (Fig. 6g).

## Discussion

The current study sought to determine the efficacy of a collagen-HS3 combination device for periodontal regeneration in a NHP periodontitis model and build on encouraging outcomes from a recently published rodent model^39^. The results show that the collagen device containing HS3 significantly promoted the regeneration of periodontal tissues, including alveolar bone, cementum, and periodontal ligament, compared to the collagen alone control. Notably, the collagen-HS3 combination device containing 180 µg HS3 (HS high) group performed equivalent, if not better, than the Emdogain group, which enables us to safely reject the null hypothesis. In general, these results suggested that the collagen-HS3 combination device has the potential to be a standalone treatment for periodontal regeneration.

The ideal treatment outcome for periodontal defects consists of a clinical absence of bleeding on probing, shallow periodontal pocket depth and no soft-tissue recession^48^. These desired clinical outcomes require rebuilding the alveolar bone and regeneration of periodontal tissues like cementum and periodontal ligament, which are vital components of the periodontium and critical to the functionality and stability of the periodontium and tooth. One major challenge in regenerating damaged/lost periodontal tissues is the lack of bone regeneration in the periodontal defect and lack of cementum and PDL regeneration on the exposed root surface, due to the rapid proliferation of gingival epithelial cells in the periodontal defect^49^. This epithelization is a defensive action to protect the periodontal defect from the oral environment, but it prevents the attachment of cells forming bone, cementum and PDL^50^. The prevention of such an epithelization and the execution of a controlled guided regeneration often necessitates the use of a barrier membrane^11^. Our data show that similar to Emdogain, the collagen-HS3 combination device significantly elevated alveolar bone formation, cementum formation and PDL formation compared to the collagen alone group, without the use of a barrier membrane.

It Is important to note that the collagen-HS3 combination device contains no exogenous growth factors or transplanted cells. Instead, a glycosaminoglycan-based strategy was used by introducing heparan sulfate to the device to help bind and mediate the activity of pro-healing endogenous growth factors at the host site, protecting them against enzymatic proteolysis^51,52^. The HS3 variant was isolated based on increased binding affinity to BMP-2 and has been shown to enhance BMP-2 mediated osteogenic activity at the injured site to support bone formation in previous studies^33,53^. In this study, our results show that the collagen-HS3 combination device not only managed to rebuild the alveolar bone socket in the periodontal defect, but also significantly improved cementum and ligament formation compared to collagen alone (*p* < 0.0001, Fig. 5). These results aligned with the observations reported in our previous rat study, where the collagen/HS3 combination device increased new alveolar bone formation and improved functional ligament attachment^39^.

Moreover, the collagen-HS3 combination device helped to reduce epithelial downgrowth, an event in which epithelial and fibrous tissues fill up the defect space before bone healing. Epithelial downgrowth exposes the defect to microbial pathogens, which may cause infection and chronic inflammation, impeding the healing process^54^. We observed considerable epithelial downgrowth in the collagen control (Fig. 5a), together with large gaps between the alveolar bone and the tooth, as well as inhibited cementum and periodontal ligament regeneration (Fig. 5c,d). These outcomes were largely alleviated with the collagen-HS3 device, possibly because heparan sulfate materials, like HS3, can regulate chemokine and cytokine gradients in the matrix^55–57^, establishing a pro-osteostimulatory environment that stimulates alveolar bone formation to fill the defect before the down growth of epithelial, so preventing complications like non-healing from occurring. However, future studies are needed to determine which endogenous growth factors bind to HS3 in vivo and potentiate periodontal regeneration through the actions of multiple tissue types.

Although protein-based therapies like Emdogain and PDGF have been deployed to treat periodontal defects in several clinically relevant animal models^18,19,58^, their overall performance was not always consistent and predictable. Emdogain has been combined with autograft or ceramics and delivered as a combination device^59,60^. Using the wire-induced intrabony defect model in NHPs, Cochran et al., showed that Emdogain improved new bone and cementum formation over a 5-month healing period^59^. A study using the same model showed that the performance of Emdogain was no better than conventional treatments like GTR^61^. Our data show that Emdogain achieved 40.4% (± 10.3%) epithelium downgrowth and 49.4% (± 5.3%) cementum regeneration (Fig. 5a,b) over a 3-month healing period. This is in line with a recent NHP study in which Emdogain achieved 47% epithelium downgrowth and 42.5% cementum regeneration^62^. In comparison, the collagen-HS3 combination device reduced the epithelium downgrowth to 36.7% (± 7.0%) (HS low) and 27.2% (± 12.7%) (HS high) while improving cementum regeneration to 60.6% (± 9.6%) (HS low) and 70.7% (± 14.4%) (HS high) respectively. These improvements are better reflected in the PRAS scores which evaluated the regeneration of lost tissue and the restoration of lost function across the entire periodontal site. The overall evaluation showed both Emdogain and HS3-containing devices were effective treatments for the periodontal defects. The unequal sample size and the loss of several specimens during processing is a limitation in this study. Despite that, HS high group is the only treatment able to promote the healing to a healthy status (score > 13) for more than half of the specimens. This demonstrates the enhanced efficacy of HS3-containing devices in this clinically relevant periodontitis model.

Comparing to the vulnerable growth factors, the HS3 material can be sterilized by gamma-irradiation without losing its ability to enhance BMP2-mediated osteogenic effect^36^. This lends HS3 to developing pre-sterilized combination devices that can be immediately deployed at the surgical site without the need for prior mixing due to incompatible sterilization methods. It also does not require cold-chain shipping or storage, given its thermostability. Therefore, the HS3 material can be effectively incorporated into medical devices and easily implemented into current surgical procedures.

To the best of our knowledge, no other scoring system similar to PRAS has been used in the dental field to evaluate the overall repair of periodontal tissue. This scoring system is based on microscopic measurements using anatomical landmarks that can be objectively identified from histological sections. Therefore, it is a standardized method with high reproducibility. Its limitation is that although the “normal” grade is well validated using clinically healthy controls but currently there is a lack of good histological examples to represent the status below “normal”. Future studies using a progressive periodontitis model would provide histological examples representing different stages of periodontal disease to finetune grades below normal.

We conclude that the collagen-HS3 combination devices promoted alveolar bone formation and the regeneration of cementum and periodontal ligament in a clinically relevant periodontitis NHP model. These findings highlight the potential of HS therapeutics for periodontal regeneration and warrant further development of this technology towards clinical applications.

## Methods

### Raw materials

CollaPlug cylinders were purchased from Zimmer Biomed (Warsaw, IN, USA). HS3 was isolated using affinity chromatography and sterilized as described previously^25^. Emdogain was purchased from Straumann (Basel, Switzerland).

### Preparation of collagen implant

Cylindrical collagen scaffolds with a diameter of 7.5 mm and a height of 5 mm were prepared from CollaPlug cylinders. Each scaffold was loaded with distilled water (150 µl) containing either 36 µg of HS3 (HS low) or 180 µg of HS3 (HS high), or no HS3 (150 µl distilled water alone) (Col). The scaffolds were then lyophilized and kept at room temperature until implantation. All steps were performed under aseptic conditions.

### In vivo evaluation

The animal model has been previously reported by Wang et al.^20^ and the study is reported in accordance with ARRIVE (Animal Research: Reporting of In Vivo Experiments) guidelines. All animal procedures were performed at the SingHealth Experimental Medicine Centre (SEMC), between 17 Nov 2016 and 21 Nov 2018, following the national guidelines for the care and use of laboratory animals, and approved by the Institutional Animal Care and Use Committee (IACUC) of Singapore Health Services (IACUC No.2016/SHS/1232, approved on 28 Sep 2016). This study used ten adult non-human primates (Macaca fascicularis) (Nafovanny, Long Thanh, Vietnam), each weighing 3.5–5.0 kg. A sample size of n = 6 was determined through a priori power assessment, considering an alpha level of 0.05 and a power of 80%, in order to detect a significant difference between the test and control groups, assuming a difference of 40%^20,39^. In the case of the Col and HS high groups, additional samples from a parallel study were combined to augment the sample size without requiring additional animals, complying with animal reduction principles and ethics. Four additional samples were used for Emdogain as the positive control. In total, this study encompassed 32 sites allocated to ten NHPs according to Latin Square design. All animals possessed full adult dentition and were healthy at the baseline checkup. The animals, housed in individual cages, were provided with water ad libitum and solid food daily, except before surgery when the animals fasted overnight. The animals were regularly checked for general health and well-being during the entire duration of the study.

### Induction of periodontitis (Month 0)

This procedure aimed to prepare the defect sites and induce plaque formation and inflammation, simulating periodontitis development. Before surgery, animals were sedated with an intramuscular (IM) injection of 15 mg/kg ketamine (Ceva; Ceva Animal Health, Gleronie, NSW, Australia) and 0.05 mg/kg atropine (Atropine Sulphate; Atlantic Laboratories, Samut Prakan, Thailand,). General anesthesia was administered and maintained via intubation of 2% isoflurane (Isothesia NXT, Piramal, Telangana, India) by a qualified veterinarian. Before surgery, local anesthesia (1 ml 2% Scandonest, Septodont, Saint-Maur-des-Fossés, France) was delivered by inferior alveolar nerve and buccal nerve block. An IM antibiotics injection of 6–8 mg/kg ampicillin (Standacillin; Sandoz, Kundl, Austria) and/or cloxacillin (Meixamr 500; PT Meiji, Jawa Timur, Indonesia) and an IM analgesics injection of 15–30 mg/kg ketorolac (Ketorolac Tromethamine; Hospira, Lake Forest, IL, USA) were given post-surgery. The operation was performed under sterile conditions. Following general and local anesthesia, the first premolars and first molars at both sides of the mandible were extracted. In total, four teeth were removed from each animal. Stainless steel wires of Ø 0.4 mm (GC orthodontics, Breckerfeld, Germany) were twisted around the neck of each experimental tooth (second premolar or second molar), and the end of the knot extended to the base of the socket of the extracted tooth (Fig. 1a). No oral hygiene measures were performed for 3 months after the wire placement. The animals were given soft diets to induce plaque accumulation around the experimental teeth and simulate the conditions for periodontitis (Fig. 1b).

### Plaque removal and hygiene establishment (Month 3)

The accumulated plaque was removed at this stage, and oral hygiene was re-established in the animal to simulate periodontal debridement in periodontitis patients. In brief, 3 months after the initial tooth extraction procedure, the wires were removed, and the root surfaces of the experimental teeth were scaled, planed, and polished using an ultrasonic scaler and a hand scaler to remove all plaque accumulation (Fig. 1c). The surgically debrided area was then irrigated with 0.2% chlorhexidine solution (Baxter, Deerfield, IL, USA). Soft food was given for a week after wire removal to facilitate healing. While it is desirable to perform daily tooth brushing on NHPs to match the oral hygiene regime of humans, it is highly challenging to do so because of the need to sedate the animal before tooth brushing. To avoid the potential detrimental effects to the animals’ health, a veterinarian performed weekly tooth brushing on each sedated animal with an electronic toothbrush to prevent plaque accumulation until the regenerative surgery at Month 4.

### Regenerative surgery and implantation (Month 4)

The periodontal defects refinement and regenerative procedures were performed 1 month after re-establishing oral hygiene. Following general and local anesthesia, the full-thickness gingival flaps were raised, and the granulation tissue was removed using scalers. Each defect was refined and standardized using a piezotome (Piezosurgery, Mectron, Carasco, Italy) to an approximate volume of 60 mm^3^, with a dimension of 3 × 4 × 5 mm^3^ (mesiodistal length × depth × buccal-lingual width). The root surfaces of the experimental teeth were debrided, and the defects were rinsed with sterile saline (B Braun, Penang, Malaysia) and dried with sterile gauze. Each defect was then implanted with one of the following: (a) CollaPlug alone (Col), (b) CollaPlug + HS low (HS3 at 36 µg), (c) CollaPlug + HS high (HS3 at 180 µg), and (d) Emdogain (Emdo). Emdogain (Straumann, Basel, Switzerland) was administrated as a gel via a syringe and needle provided by the manufacturer. The Collaplug scaffolds were slightly compressed to fit snuggly into the defect. After implantation, the defects were closed using resorbable sutures (Vicryl 5–0, Ethicon, Bridgewater, NJ, USA) (Fig. 1d). After surgery, antibiotics and analgesics were given as described above, and the animals were closely monitored. Soft food was given for a week postoperatively to facilitate healing.

### Sacrifice and harvest (Month 7)

Three months after the regenerative surgery, the animals were euthanized. First, the animals were anesthetized with an IM injection of 15 mg/kg ketamine and a subcutaneous injection of 0.05 mg/kg atropine. Then the animals were euthanized by an IV injection of 100 mg/kg pentobarbital (Valabarb; Jurox, Maitland, NSW, Australia). Next, the soft and hard tissues surrounding the defects were harvested together with the experimental teeth (Fig. 1e), and the tissue blocks were immediately fixed in 10% neutral buffered formalin (Sigma-Aldrich, Burlington, MA, USA).

Clinical assessment of the health of the periodontal tissues.

To track and monitor the health of the periodontal tissues during the study, clinical assessment for periodontal indices, including plaque index (PI), number of bleeding points on probing (BoP), gingival inflammation (GI) and periodontal probing depth (PPD), was performed and recorded at the following timepoints: (i) before teeth extraction (Month 0), (ii) before wire removal (Month 3), (iii) before regenerative surgery (Month 4), (iv) one (1) month after regenerative surgery (Month 5), and (v) before sacrifice (Month 7) regardless of treatment groups. These non-invasive periodontal measurements were part of a routine clinical examination to monitor periodontitis status. The measurements were performed by a qualified dental surgeon at 6 locations around the tooth: mesial-buccal (mb), mid-buccal (mid-b), distal-buccal (db), distal-lingual (dl), mid-lingual (mid-l), and mesial-lingual (ml) (Supplementary Fig. 2). PI is a visual evaluation with 4 scores, from [0 = absence of plaque accumulation] to [3 = severe plaque accumulation]. BoP is a 2-score measurement, with [0 = no bleeding] and [1 = bleeding]. GI is a visual evaluation with a range of 4 scores [0 = absence of inflammation to 3 = severe inflammation characterized by marked redness, swelling and tendency to bleed]^63,64^. PPD, measured with a periodontal probe, is the distance from the gingival margin to the bottom of the periodontal pocket and quantifies the loss of attachment between the periodontium and the tooth. Detailed information about the clinical assessment measurements is shown in Supplementary Table 2.

### Micro-CT imaging

The samples were scanned using ex vivo micro-computed tomography (µCT). Formalin-fixed explants were scanned using a Skyscan 1176 micro-CT scanner (Bruker, Kontich, Belgium) at a resolution of 35 µm, a voltage of 65 kV and a current of 385 µA. Reconstruction and analysis of scanned samples were performed using the manufacturer's software (nRecon- v1.6.9.18, DataViewer -v1.5.4.0, cTvox-v3.0.0 r1122, Bruker, Kontich, Belgium).

Histology.

**Figure 7:**
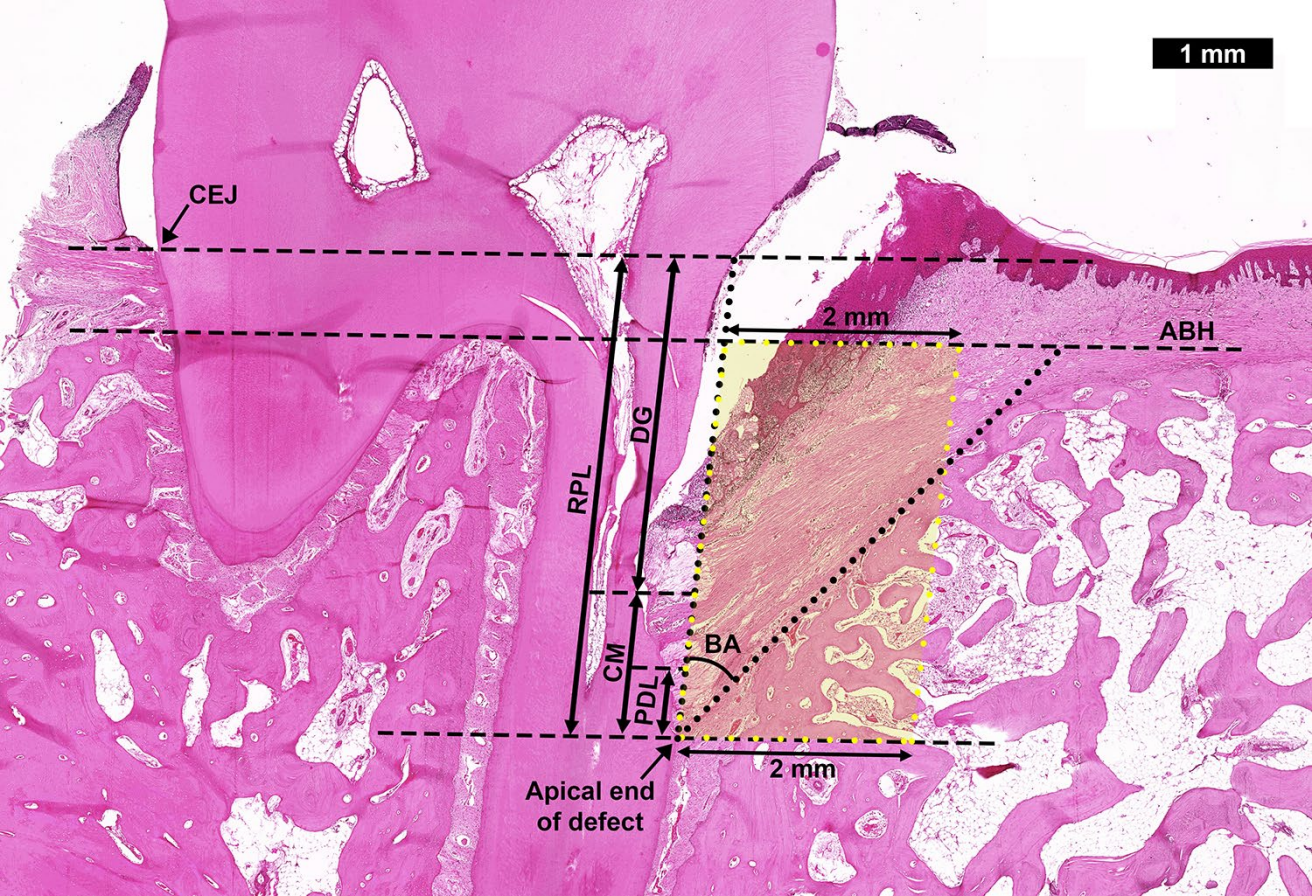
Representative image demonstrating the microscopic measurements. The landmarks for the measurements, i.e. cementum-enamel junction (CEJ), alveolar bone height (ABH) and apical end of defect, are marked with straight dashed lines. For the principal measurements, the lengths of root plane surface (RPL), epithelial downgrowth (DG), cementum regeneration (CM) and periodontal ligament regeneration (PDL) are represented with arrows. The bone angle (BA) is the angle between the RPL and the surface of the regenerated alveolar bone, represented with dotted lines. The region of interest (ROI) for quantitative bone histomorphometry is represented with a yellow-shaded box.

After micro-CT imaging, the samples were decalcified in Osteosoft (Merck, Darmstadt, Germany) on an orbital shaker for at least 2 months. The samples were divided mesiodistally into two halves and subsequently dehydrated in a graded series of ethanol (70–100%). After infiltration with xylene and embedding in paraffin, the samples were sectioned with a rotary microtome (RM2255, Leica, Wetzlar, Germany) in an occlusal-apical direction along the mesiodistal plane, with a thickness of 5 µm per section. The sections were collected on Polysine slides, dried at 60 °C for 1 h, and stained with hematoxylin and eosin (Merck, Darmstadt, Germany) and trichrome (Abcam, Cambridge, United Kingdom). After the stained slides were mounted and cleaned, they were viewed and scanned using a microscope (Zeiss Axio Imager Z2, Zeiss, Oberkochen, Germany) equipped with MetaSystem stage control and the Metafer4 software (MetaSystems, Altlussheim, Germany). For histomorphometric analysis, three sections ~ 50 µm apart were selected. Histomorphometry was done using the histogram function in Adobe Photoshop (version 21.2.4, Adobe, San Jose, CA, USA) to evaluate the new bone formation in the region of interest (ROI) (Fig. 7). The ROI is defined as the area between the line from the alveolar bone height (ABH), the apical end of the root-planed defect, and a parallel line of 2 mm. New bone formation is determined as BV/TV% = *pixels of bone* × 100/*pixels of ROI*. To reflect the volume of bone supporting the teeth, (BV + MV)/TV%, which included both the volume of bone trabeculae and bone marrow, was also determined as (BV + MV)/TV% = (*pixels of bone* + *pixels of bone marrow*) × 100/*pixels of ROI*. The histomorphometric bone analysis was taken from the average value of 3 slides per sample.

### Histological analysis

The regenerative effect of each treatment was further quantified using the linear measurement function in Photoshop, according to the analysis method reported by Wang et al.^20^ First, the root-planed length (RPL) was defined as the distance between the cementum-enamel junction (CEJ) and the apical tip of the created defect (Fig. 7). Thereafter, the following parameters were defined and measured:

1. Epithelium downgrowth (DG, expressed in %): [*distance between CEJ and apical junction of epithelium*] / *RPL* × 100%
2. Cementum regeneration (CM, expressed in %): [*distance between the apical end of root-planed margin and coronal extent of cementum tissue on the denuded root surface*] / *RPL* × 100.
3. PDL regeneration (PDL, expressed in %): [*distance between the apical and coronal extent of ligament structure attached to the root surface*] / *RPL* × 100.
4. Bone angle (BA): *Angle between root-planed surface and plane of newly formed alveolar bone*.

The DG, CM, PDL and BA measurements indicate the extent of regeneration of periodontal tissues and soft tissue infiltration into the defect. As the native periodontal tissues were removed during defect creation, any periodontal tissues observed in the defect area post-surgery were considered newly regenerated. Epithelial downgrowth is the extension of epithelial tissue invading the space in the periodontal defect. The invasion of epithelial tissue prevents the attachment of periodontal tissues to the root surface and, therefore, should be minimized or avoided. The cementum and periodontal ligament are essential components of the periodontium, and their regenerations contribute to the stability of the tooth in the alveolar socket. The new PDL formation was defined as the development of fibers perpendicularly oriented to the root surface and embedded between the newly formed cementum and bone. The bone angle indicates the extent of alveolar bone regeneration. A smaller bone angle means closer contact between the tooth and the alveolar bone and greater stability of the tooth within the alveolar socket.

Three slides per specimen were used for these measurements, and the average value was used for statistical analysis. For samples where all three sections show a complete root and intact periodontium on the distal side of the 2nd premolar/molar, measurements were also made on the distal root surface and adjacent periodontium. This set of measures at the uninjured side of the tooth was compiled to reflect the “healthy control” condition, which would be used to indicate the extent of regeneration at the defect site towards its original status.

### Periodontal regeneration assessment score (PRAS)

Considering DG and BA values are negatively associated with regeneration, while CM and PDL values are positively associated with improved regeneration, and they are expressed in either percentages or degrees, we converted the histological measurements into a matrix score using a standardized scoring system PRAS. Using a scoring rubric, the DG, CM, PDL and BA measurements were converted from percentages or degrees into a 5-point scales, following a linear interpolation principle (Table 1). The individual scores were then summed to form a total PRAS score which collectively quantifies the efficacy of each treatment. Depending on the total PRAS score, the extent of healing was graded from “severely abnormal” to “normal” level. Beside the experimental groups, the DG, CM, PDL and BA values of the 15 healthy samples were also measured and assessed to validate the PRAS scoring system.

**Table 1:** Periodontal regeneration assessment score (PRAS).

PRAS	Score
Epithelial downgrowth (DG)	
Less than 20%	4
21–40%	3
41–60%	2
61–80%	1
Above 80%	0
Cementum regeneration (CM)	
More than 80%	4
61–80%	3
41–60%	2
21–40%	1
Less than 20%	0
Periodontal ligament regeneration (PDL)	
More than 80%	4
61–80%	3
41–60%	2
21–40%	1
Less than 20%	0
Bone angle (BA)	
Full integration between host bone and tooth (< 5°)	4
Less than 15°	3
16–30°	2
31–45°	1
> 45°	0
Overall regeneration assessment	
Grade I: normal	13–16
Grade II: nearly normal	9–12
Grade III: abnormal	5–8
Grade IV: severely abnormal	0–4

### Statistical analyses

Statistical analysis was carried out by one-way ANOVA followed by Tukey's multiple comparisons post hoc testing using GraphPad Prism (Version 9.2.0, Dotmatics, San Diego, CA, USA). A *p-value* < 0.05 is considered statistically significant. *: *p* < 0.05, **: *p* < 0.01, ***: *p* < 0.001, ****: *p* < 0.0001.

## Supplementary Information

Supplementary Figure 1.

Supplementary Figure 2.

Supplementary Table 1.

Supplementary Table 2.

## Data Availability

Original data are available from the corresponding author upon reasonable request.
