# Supplementary material for: Affinity-selected heparan sulfate collagen device promotes periodontal regeneration in an intrabony defect model in Macaca fascicularis
Source: Sci Rep. 2023 Jul 21;13:11774. doi: 10.1038/s41598-023-38818-y (PMC10362032; doi:10.1038/s41598-023-38818-y)
Supplement: Supplementary file 1 — Supplementary Figure 1. [file 41598_2023_38818_MOESM1_ESM.pdf]

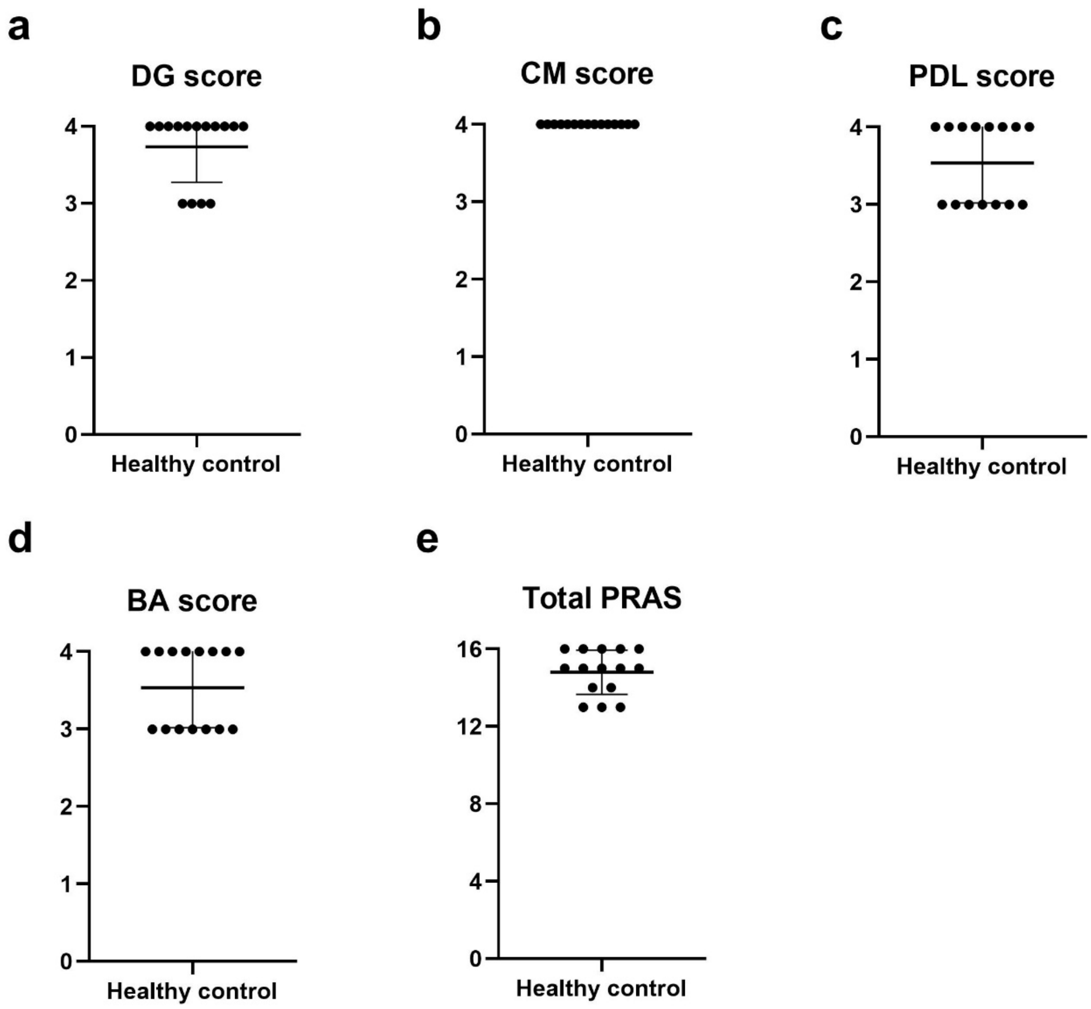

Supplementary Figure 1: PRAS score of healthy controls. A: DG score; B:CM score; C: PDL score; D: BA score; E: Total PRAS score
