# Supplementary material for: Affinity-selected heparan sulfate collagen device promotes periodontal regeneration in an intrabony defect model in Macaca fascicularis
Source: Sci Rep. 2023 Jul 21;13:11774. doi: 10.1038/s41598-023-38818-y (PMC10362032; doi:10.1038/s41598-023-38818-y)
Supplement: Supplementary file 2 — Supplementary Figure 2. [file 41598_2023_38818_MOESM2_ESM.pdf]

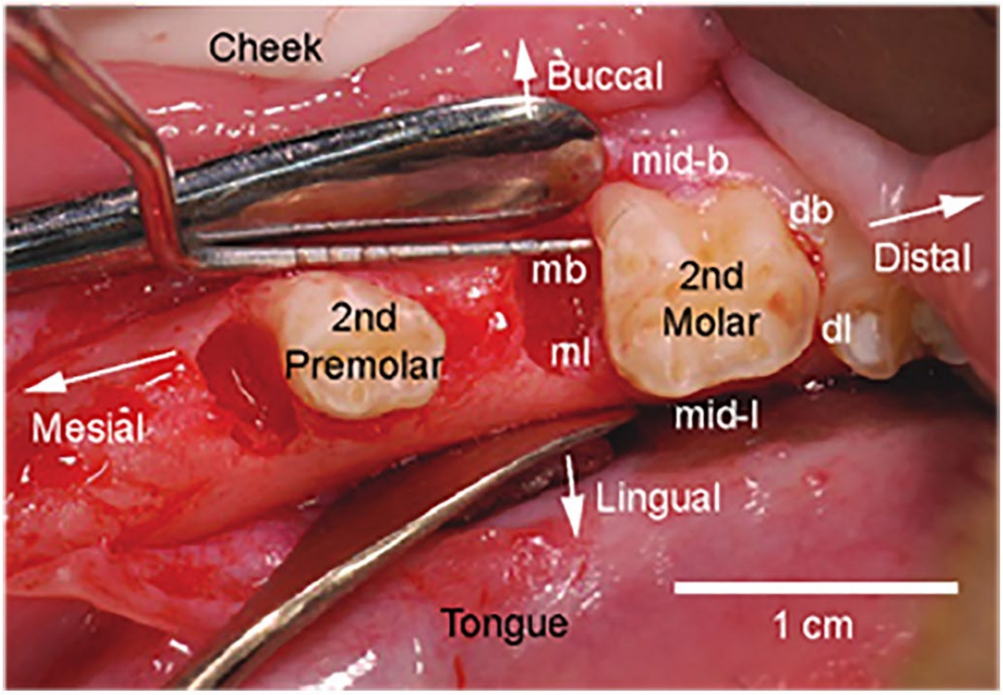

Supplementary Figure 2: Overview of the surgery site, with the landmarks of the clinical assessment labelled. For each tooth, the clinical measurements were performed by a qualified dental surgeon at 6 locations: mesial-buccal (mb), mid-buccal (mid-b), distal-buccal (db), distal-lingual (dl), mid-lingual (mid-l), and mesial-lingual (ml)
