# Supplementary material for: Affinity-selected heparan sulfate collagen device promotes periodontal regeneration in an intrabony defect model in Macaca fascicularis
Source: Sci Rep. 2023 Jul 21;13:11774. doi: 10.1038/s41598-023-38818-y (PMC10362032; doi:10.1038/s41598-023-38818-y)
Supplement: Supplementary file 3 — Supplementary Table 1. [file 41598_2023_38818_MOESM3_ESM.pdf]

Supplementary Table 1: Animal usage table

Animal		Tooth Position	Test group	Micro-CT	Histology	Histological Analysis
1 (ID 815)	Left	Pre-Molar	Used in a parallel study			
	Left	Molar	Col + HS Low	✓	✓	✓
	Right	Pre-Molar	Col + HS High	✓	✓	✓
	Right	Molar	Used in parallel study			
2 (ID 816)	Left	Pre-Molar	Col + HS Low	✓	✓	X
	Left	Molar	Col + HS High	✓	✓	✓
	Right	Pre-Molar	Used in a parallel study			
	Right	Molar	Col	✓	✓	✓
3 (ID 843)	Left	Pre-Molar	Col + HS Low	✓	✓	✓
	Left	Molar	Col + HS High	✓	✓	✓
	Right	Pre-Molar	Used in parallel study			
	Right	Molar	Emdo	✓	✓	✓
4 (ID 846)	Left	Pre-Molar	Col	✓	✓	✓
	Left	Molar	Emdo	✓	✓	✓
	Right	Pre-Molar	Col + HS High	✓	✓	✓
	Right	Molar	Col + HS Low	✓	✓	✓
5 (ID 2452)	Left	Pre-Molar	Used in parallel study			
	Left	Molar	Col	✓	✓	✓
	Right	Pre-Molar	Emdo	✓	✓	✓
	Right	Molar	Used in parallel study			
6 (ID 5293)	Left	Pre-Molar	Emdo	✓	✓	✓
	Left	Molar	Used in parallel study			
	Right	Pre-Molar	Col + HS Low	✓	✓	✓
	Right	Molar	Col + HS High	✓	✓	✓
7 (ID 7330)	Left	Pre-Molar	Col + HS High	✓	✓	✓
	Left	Molar	Used in parallel study			
	Right	Pre-Molar	Col	✓	✓	X
	Right	Molar	Col + HS Low	✓	✓	✓
8 (ID 2549)	Left	Pre-Molar	Col + HS High	✓	✓	✓
	Left	Molar	Col	✓	✓	✓
	Right	Pre-Molar	Col	✓	✓	✓
	Right	Molar	Col + HS High	✓	✓	✓
9 (ID 8152)	Left	Pre-Molar	Col	✓	✓	✓
	Left	Molar	Col + HS High	✓	✓	✓
	Right	Pre-Molar	Col + HS High	✓	✓	✓
	Right	Molar	Col	✓	✓	✓
10 (ID 8348)	Left	Pre-Molar	Col + HS High	✓	✓	X
	Left	Molar	Col	✓	✓	✓
	Right	Pre-Molar	Col	✓	✓	✓
	Right	Molar	Col + HS High	✓	✓	✓
			Total	32	32	29
