# Supplementary material for: Affinity-selected heparan sulfate collagen device promotes periodontal regeneration in an intrabony defect model in Macaca fascicularis
Source: Sci Rep. 2023 Jul 21;13:11774. doi: 10.1038/s41598-023-38818-y (PMC10362032; doi:10.1038/s41598-023-38818-y)
Supplement: Supplementary file 4 — Supplementary Table 2. [file 41598_2023_38818_MOESM4_ESM.pdf]

Supplementary Table 2: Scoring system for clinical assessment

Clinical assessment	Score
Plaque Index (PI)<br>● No plaque present<br>● Plaque present on tooth surface near the CEJ<br>● Plaque covering less than half of tooth surface from CEJ to apical extent<br>● Plaque extending over the entire tooth surface and covering more than half the tooth surface from CEJ to the apical extent	 0<br>1<br>2<br>3
Bleeding on probing (BoP)<br>● No bleeding<br>● Bleeding	 0<br>1
Gingival inflammation index (GI)<br>● Absence of inflammation<br>● Mild to moderate inflammation at localized spot(s)<br>● Mild to moderately severe inflammation extending over a wider area<br>● Severe inflammation characterized by marked redness, swelling and a tendency to bleed	 0<br>1<br>2<br>3
Probing depth (PPD) - A measurement from the gingival margin to the bottom of the periodontal pocket	Depth (mm)
